# Pt-Rare Earth Subnanometric Bimetallic Clusters Efficiently Catalyze the Reverse Water–Gas Reaction

**DOI:** 10.3390/nano16010077

**Published:** 2026-01-05

**Authors:** Zhaolei Liang, Chang Sun, Songhe Shen, Qingqing Li, Feng Luo

**Affiliations:** 1 Tianjin Key Laboratory for Rare Earth Materials and Applications, Center for Rare Earth and Inorganic Functional Materials, School of Materials Science and Engineering, National Institute for Advanced Materials, Nankai University, Tianjin 300350, China; liangzhaolei@mail.nankai.edu.cn (Z.L.); sunchang@mail.nankai.edu.cn (C.S.); 2 Department of Physics, University of California at Santa Barbara, Santa Barbara, CA 93106, USA; shen16052061@gmail.com; 3 College of Renewable Energy, Hohai University, Nanjing 211100, China

**Keywords:** subnanometric bimetallic clusters, platinum-rare earth clusters, reverse water gas shift reaction, synergistic effect

## Abstract

The reverse water–gas shift (RWGS) reaction serves as a highly flexible and critical pathway for converting CO_2_ into CO, with Pt-based catalysts having been widely investigated. Here, a series of platinum-rare earth (RE) subnanometric bimetallic clusters (SBCs) were successfully prepared on carbon support by the potassium vapor reduction method. Their structure and electronic properties, along with catalytic performance, were systematically characterized and evaluated. The Pt-RE SBC catalysts exhibited excellent catalytic activity, maintaining CO selectivity above 95% at high CO_2_ conversion levels and demonstrating stable operation over 100 h at 600 °C. Furthermore, the influence of different supports (carbon black and CeO_2_) on the catalytic performance was compared. It was found that Pt-Sc SBCs supported on the carbon exhibited better dispersion, smaller particle size, and superior catalytic performance relative to the CeO_2_ supported counterpart. This study provides new insights into the design of highly efficient and stable RWGS catalysts, highlighting the key role of the Pt-RE SBC interface synergistic effect and support selection, which is of great significance for the resource utilization of CO_2_.

## 1. Introduction

With the ongoing consumption of fossil fuels, atmospheric CO_2_ concentrations continue to increase, leading to increasingly serious global greenhouse effects and adverse outcomes such as ocean acidification [1,2]. Carbon capture and utilization (CCU) serves as an effective solution, aiming to mitigate the challenges associated with CO_2_ emissions. The core of CCU lies in converting captured CO_2_ into high-value-added products through chemical synthesis, thereby enabling carbon recycling and emission reduction [3,4]. Among the various approaches, the conversion of CO_2_ to CO via the reverse water–gas shift (RWGS) reaction represents a highly flexible and critical pathway [5]. The produced CO acts as a vital platform molecule, which can be further converted into high-value chemicals such as olefins and alcohol-based fuels through the Fischer–Tropsch (F-T) synthesis process. This integrated route holds significant importance for environmental improvement and transition of future energy systems, attracting widespread attention [6,7].

The high bond energy and thermodynamic stability of the C=O bond in a CO_2_ molecule necessitates substantial energy input for its direct activation, while the kinetic limitations further restrict the reaction rate. Therefore, it is very important to develop efficient catalysts [8]. Catalysts prepared by supporting active metals on inorganic oxides (e.g., SiO_2_ [9], Al_2_O_3_ [10], TiO_2_ [11], or CeO_2_ [12,13]) have demonstrated high efficiency. Based on the active metal, these catalysts can be classified into non-noble metals (e.g., Cu [14,15], Ni [16,17], Co [18]) and noble metals (e.g., Pt [9,19], Pd [20], Ru [21,22]). Among these active metals, Cu, Ni, and Co exhibit low CO desorption energy, which helps inhibit methanation to some extent. However, these catalysts often suffer from limited thermal stability under operating conditions with an appreciable CO_2_ conversion rate. In contrast, noble metal catalysts (e.g., Pt, Pd, Ru) achieve higher CO_2_ conversion at low temperature, yet their stronger CO adsorption energy tends to promote CH_4_ formation during the reaction [19,23,24]. Therefore, further study is still needed to improve CO selectivity and catalyst stability while maintaining high CO_2_ conversion rate.

Pt-based catalysts are widely employed in RWGS reactions due to their high conversion at low temperatures. Considerable efforts have been devoted to the design and preparation of Pt-based catalysts, with strategies such as improving metal dispersion [12], modifying supports via doping [25], and constructing alloy structures [26,27,28,29,30,31] proving effective in enhancing catalytic performance. Among these, the formation of Pt with other metals offers a promising approach to adjust the electronic properties, thereby significantly improving CO_2_ conversion and CO selectivity while maintaining excellent stability at high-temperature conditions. Compared with monometallic Pt/SiO_2_ catalysts, the Pt-In/SiO_2_ catalyst exhibited near 100% the CO selectivity and higher activity in the temperature range of 140–200 °C [26]. Li et al. [28] used density functional theory (DFT) and Kinetic Monte Carlo (KMC) simulations to demonstrate that supported Pt-Ni alloy nanostructures outperform their monometallic Pt or Ni counterparts during RWGS reactions. Zhang et al. [29] further explored the synergistic mechanism between Ni and Pt sites on Pt_3_Ni nanowires during RWGS reactions. They identified a unique Ni-Pt hybrid configuration at the edges of nanowires, which contributed to high catalytic activity. In a structural design study, a PtFe core–shell catalyst was prepared (Pt/Fe = 1:1) and its catalytic activity was 5.1 times higher than that of Pt nanoparticles, highlighting the effectiveness of bimetallic synergy core–shell architecture in thermal catalytic process [30]. Moreover, a dual-site catalyst comprising Pt and Fe atoms co-loading on the surface of CeO_2_ was reported [31]. In this system, Fe atoms not only act as electronic promoters to modulate the charge density of Pt but also suppress CO methanation, thereby enhancing the catalytic activity and stability.

Rare earth (RE) elements, are often referred to as “industrial vitamins”, play a vital role in various industrial applications. In catalysis, they have been widely utilized in processes such as propane dehydrogenation and ethane oxidative dehydrogenation (EOD) [32,33,34]. Pt-M (M = La, Y and Sc) nanoparticles supported on zeolite nanosponges were synthesized and evaluated in the oxidative dehydrogenation of ethane using CO_2_ as a soft oxidant [33]. Ryong Ryoo et al. [34] reported that PtY intermetallic compound nanoparticles formed within mesoporous zeolite serve as a highly active, selective and stable catalyst for propane dehydrogenation. Due to its unique electronic structure, rare earth alloys can improve the catalytic activity by regulating the electronic structure and d-band center of active metals [34,35,36]. Sun et al. [36] synthesized a series of Pt-Ru-RE ternary alloys exhibiting high activity and high stability. DFT calculations showed that rare earth atoms induce a downshift in the d-band center of Pt surfaces and promote an electron-rich state, and thereby catalytic performance. Moreover, the high formation enthalpy of Pt-RE alloys leads to strong bonding between Pt-RE, thus significantly enhancing the durability of the catalysts [37]. However, the low reduction potential (ranging from −2 V to −3 V) of rare earth elements poses a challenge for their synthesis [34,38,39]. Luo et al. [36,38,39] developed a universal synthesis route based on sodium vapor reduction, and successfully realized the controllable synthesis of a series of noble metal–rare earth alloy materials. However, this strategy still has some limitations when applied to platinum-based alloy systems. An ideal carbon support should not only stabilize highly dispersed metal nanoparticles but also, through fine-tuning of its physical structure and chemical properties, synergize with the metal to collectively promote the efficient and highly selective activation and conversion of CO_2_ molecules [40]. Sub-nanometer catalysts fundamentally alter the activation mode of CO_2_ molecules by maximizing the exposure of unsaturated active atoms, inducing abrupt changes in electronic structure due to quantum size effects, and forming strong, tunable electronic interactions with the support [41].

Inspired by these advances, this work introduces a potassium vapor reduction method for preparing Pt-RE SBCs catalysts for RWGS reaction. Seven different Pt-RE SBCs (RE = La, Nd, Gd, Ho, Dy, Yb, Sc) were synthesized on carbon support. Among them, Pt-Sc SBCs nanoparticles show the highest CO_2_ conversion and CO selectivity. It was observed that a rare earth oxide layer is formed on the surface of the Pt-RE SBCs catalyst, endowing the catalyst with high conversion, high selectivity, and remarkable stability at high temperatures. In addition, the effect of catalyst support was systematically investigated. Pt-Sc SBCs supported on CeO_2_ showed inferior performance compared to the carbon-support system, primarily due to the increase in particle size and non-uniform distribution of the SBC particles on the non-carbon support. This work provides valuable guidance for the design of more effective SBC catalysts for CO_2_ conversion.

## 2. Materials and Methods

### 2.1. Materials

Chloroplatinic acid hexahydrate (H_2_PtCl_6_·6H_2_O, 99.9%) was purchased from Adamas Reagent Company, Shanghai, China, while the rare earth chloride salts hydrate (LaCl_3_·6H_2_O, NdCl_3_·7H_2_O, GdCl_3_·6H_2_O, DyCl_3_·6H_2_O, HoCl_3_·6H_2_O, YbCl_3_·6H_2_O, 99% ScCl_3_·6H_2_O) and the sodium hydroxide (NaOH, 99.999%) were purchased from Aladdin Reagent (Shanghai, China). Analytically pure potassium metal (K) was purchased from Tianjin Bohua Chemical Reagent Company, Tianjin, China. Methanol (CH_3_OH) and isopropanol (C_3_H_8_O) were purchased from Aladdin Reagent Company. Carbon black BP 2000 (Black Perals 2000) was purchased from Cabot Company, Boston, MA, USA. Ce(NO_3_)_2_·6H_2_O was purchased from Adamas Reagent Company. The water used in the experiments was lab-prepared ultrapure water (18.2 MΩ·cm).

### 2.2. Catalyst Preparation

To prepare the 1 wt% Pt-RE/C catalyst, 0.0103 mmol H_2_PtCl_6_·6H_2_O and 0.0428 mmol rare earth chloride salts (LaCl_3_·6H_2_O, NdCl_3_·6H_2_O, GdCl_3_·6H_2_O, DyCl_3_·6H_2_O, HoCl_3_·6H_2_O, YbCl_3_·6H_2_O, 99% ScCl_3_·6H_2_O) were dissolved in 20 mL of ultrapure water in a round bottom flask. Then, 200 mg of carbon black was added to the mixture, followed by sonication for 30 min, and stirring for 12 h. Following rotary evaporation and drying of the resultant mixture, the precursor powder was obtained. Within a glove box, the precursor was placed into a circular BN crucible, which was then sealed together with another BN crucible containing a K ingot inside a cuboid BN crucible. The sealed setup was heated at 600 °C (10 °C/min ramp) for 2 h in a Muffle oven. After naturally cooling to room temperature, the sample was transferred out of the glove box and washed sequentially with isopropanol, ethanol, and deionized water. To remove rare earth oxides, the sample was treated with 0.5 M H_2_SO_4_ and then vacuum dried at 60 °C for 12 h to obtain the Pt-RE/C catalyst.

### 2.3. Catalytic Performance Testing

The catalytic performance was tested in a fixed bed reactor under atmospheric pressure. A 20 mg sieved sample (20–40 mesh) was mixed with 500 mg inert SiO_2_, and loaded into a quartz tube. Prior to the reaction, the catalyst was pretreated with 5% H_2_/Ar (30 mL/min) at 600 °C for 0.5 h. The temperature of the catalyst was decreased to room temperature, and the gas flow was switched to RWGS reaction gas mixture (23% CO_2_, 69% H_2_, 8% N_2_) at a gas hourly space velocity (GHSV) of 200,000 mL·g_cat_^−1^h^−1^. The reaction was maintained for at least 1 h at each target temperature. Catalyst stability was assessed under continuous flow at 600 °C using the same GHSV and reaction atmosphere over an extended period. The outlet products were analyzed by an online gas chromatograph equipped with a thermal conductivity detector (TCD). The gas flow rate was determined by the internal standard method, with N_2_ as the internal standard. The CO_2_ conversion and the CO selectivity were calculated according to the following formula.

(1) $$X_{{\mathrm{CO}}_{2}}(\%)=\frac{n_{{\mathrm{CO}}_{2}}^{\mathrm{in}}\;-\;n_{{\mathrm{CO}}_{2}}^{\mathrm{out}}}{n_{{\mathrm{CO}}_{2}}^{\mathrm{in}}}\times 100\%=\left(1\;-\;\frac{A_{{\mathrm{CO}}_{2}}^{\mathrm{out}}/A_{N_{2}}^{\mathrm{out}}}{A_{{\mathrm{CO}}_{2}}^{\mathrm{in}}/A_{N_{2}}^{\mathrm{in}}}\right)\;\times \;100\%$$

where $n_{{\mathrm{CO}}_{2}}^{\mathrm{in}}$ is the concentration of CO_2_ in the reaction gas, and $n_{{\mathrm{CO}}_{2}}^{\mathrm{out}}$ is the concentration of CO_2_ in the outlet gas. $A_{{\mathrm{CO}}_{2}}^{\mathrm{in}}$ and $A_{N_{2}}^{\mathrm{in}}$ refer to the chromatographic peak area of CO_2_ and N_2_ in the inlet gas, respectively, $A_{{\mathrm{CO}}_{2}}^{\mathrm{out}}$ and $A_{N_{2}}^{\mathrm{out}}$ refer to the chromatographic peak area of CO_2_ and N_2_ in the outlet gas respectively. The chromatographic peak area of each component is proportional to the concentration of each component.

The calculation process of CO selectivity is as follows:

(2) $$S_{\mathrm{CO}}\left(\%\right)\;=\;\frac{n_{\mathrm{CO}}^{\mathrm{out}}}{n_{\mathrm{CO}}^{\mathrm{out}}\;+\;n_{{\mathrm{CH}}_{4}}^{\mathrm{out}}}\;\times \;100\%\;=\;\frac{A_{\mathrm{CO}}^{\mathrm{out}}\;\times \;f_{\mathrm{CO}/N_{2}}}{A_{\mathrm{CO}}^{\mathrm{out}}\;\times \;f_{\mathrm{CO}/N_{2}\;}+{\;A}_{{\mathrm{CH}}_{4}}^{\mathrm{out}}\;\times \;f_{{\mathrm{CH}}_{4}/N_{2}}}\;\times \;100\%$$

where $n_{\mathrm{CO}}^{\mathrm{out}}\;$ and $n_{{\mathrm{CH}}_{4}}^{\mathrm{out}}$ indicate the concentration of CO and CH_4_ in the gas, respectively. $f_{\mathrm{CO}/N_{2}}$ and $f_{{\mathrm{CH}}_{4}/N_{2}}$ are the relative correction coefficients of CO to N_2_ and CH_4_ to N_2_, respectively, which are calibrated by standard gas. $A_{\mathrm{CO}}^{\mathrm{out}}$ and $A_{{\mathrm{CH}}_{4}}^{\mathrm{out}}$ are the chromatographic peak areas of CO and CH_4_ detected by TCD in the outlet gas.

### 2.4. Catalyst Characterization

The crystalline phase and structure of the material were characterized by X-ray diffractometer (XRD) on a Rigaku Smart Lab diffractometer using Cu Kɑ radiation (λ = 1.5418 Å) operated at 40 kV and 40 mA. Data were collected in the 2θ range of 10° to 90° with a scanning speed of 8° min^−1^. The morphology and microstructure of the material were obtained using a transmission electron microscope (TEM) on a JEM-2800 instrument and spherical aberration corrected TEM on JEM-ARM200F instrument, JEOL Ltd., Tokyo, Japan. For TEM analysis, the sample was dispersed in ethanol and ultrasonicated for 15 min. A drop of the resulting suspension was then deposited onto a copper grid and dried at ambient temperature prior to observation. The surface chemical states and electronic structure of the catalysts were investigated using X-ray photoelectron spectroscopy (XPS). XPS measurements were conducted on a Thermo Scientific K-Alpha spectrometer (Thermo Fisher Scientific, Waltham, MA, USA) with a monochromatic Al Kɑ X-ray source (hʋ = 1486.6 eV).

## 3. Results and Discussion

**Figure 1:**
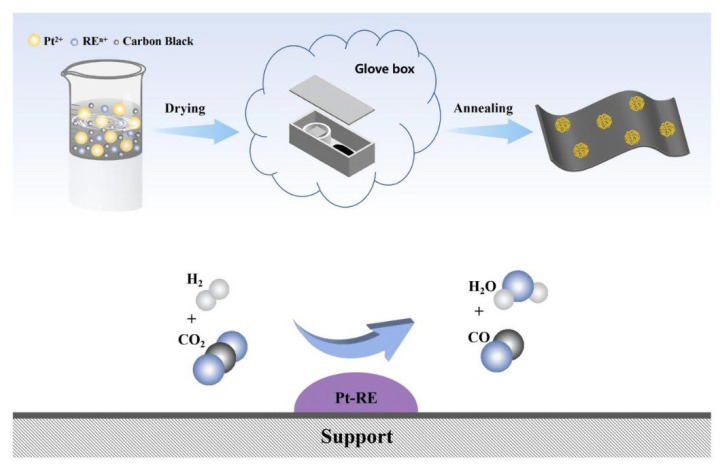
Schematic of the synthetic procedure for Pt-RE SBCs and the diagram of RWGS reaction process.

A series of Pt-RE SBCs supported on carbon were synthesized by a potassium vapor-assisted method. Figure 1 shows the schematic of the synthetic procedure for Pt-RE SBCs and the RWGS reaction process.

**Figure 2:**
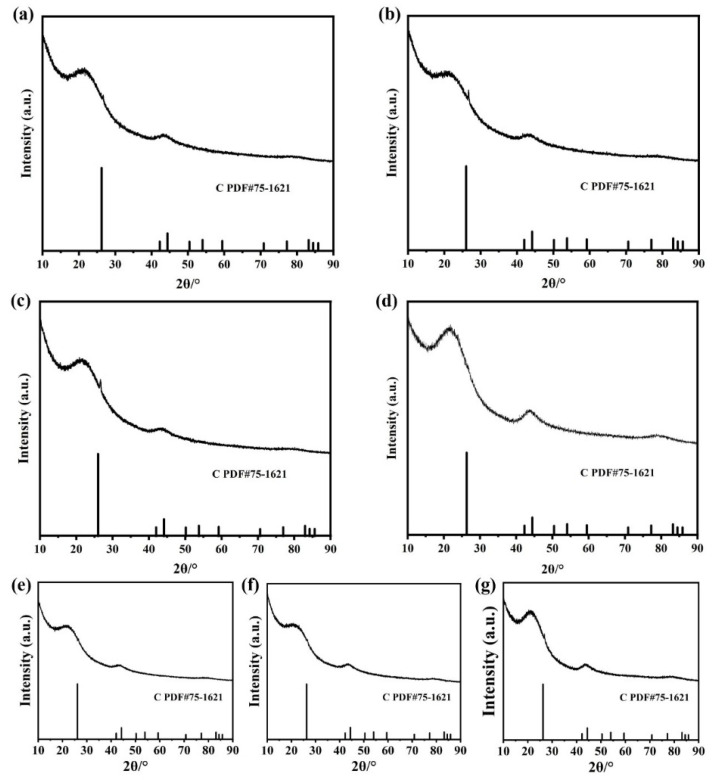
XRD patterns of (**a**) Pt-La/C, (**b**) Pt-Nd/C, (**c**) Pt-Gd/C, (**d**) Pt-Dy/C, (**e**) Pt-Ho/C, (**f**) Pt-Yb/C, and (**g**) Pt-Sc/C.

As shown in the XRD patterns (Figure 2), no detectable diffraction peaks corresponding to Pt-La, Pt-Nd, Pt-Gd, Pt-Dy, Pt-Ho, Pt-Yb, or Pt-Sc alloy phase were detected in any of the Pt-RE/C samples (Figure 2a–g). This suggests that the Pt-RE SBCs species are highly dispersed on the carbon support. Energy-dispersive X-ray spectroscopy (EDS) mapping (Figure S1a–g) further confirms the homogeneous distribution of Pt and the respective rare earth elements within individual clusters, supporting the formation of SBCs structures.

To investigate the influence of the support on the catalytic behavior of Pt-RE alloys, Pt-Sc alloys supported on CeO_2_ were also prepared. Figure S2 shows the XRD patterns of Pt-RE SBCs on different supports, which showed that no obvious diffraction peak was observed in the XRD pattern of Pt-Sc/C and Pt-Sc/CeO_2_, due to the low content and highly dispersed nature of Pt-Sc clusters/nanoparticles. EDS mapping (Figure S3d–i) further verifies the uniform co-distribution of Pt and Sc in both Pt-Sc/C and Pt-Sc/CeO_2_ catalysts.

**Figure 3:**
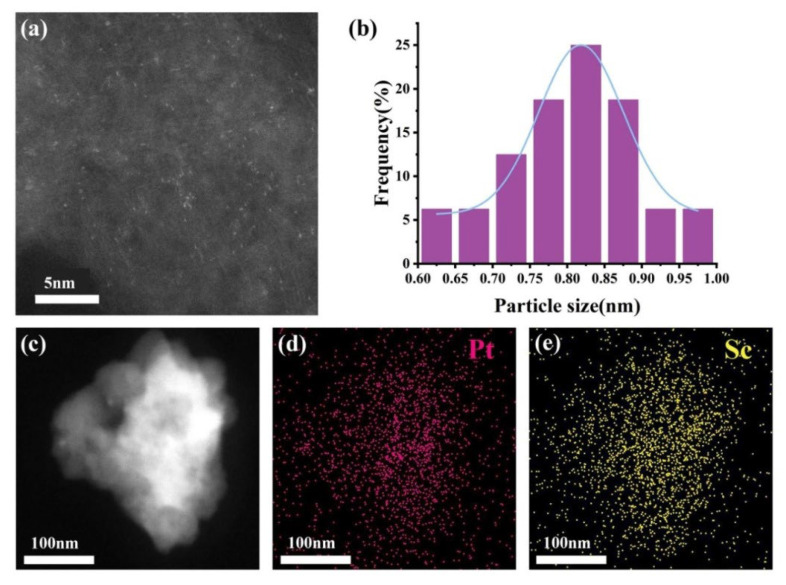
(**a**) STEM images of Pt-Sc/C, (**b**) the size distribution of Pt-Sc/C, (**c**–**e**) and the EDS elemental mapping images of Pt-Sc/C.

The morphology and cluster size distribution of the Pt-Sc SBCs catalyst was analyzed by TEM (Figure 3). The catalyst exhibits uniform dispersion of clusters on the carbon support without notable agglomeration, underscoring the efficacy of the synthetic approach. The cluster size distributions are narrow, predominantly falling in the range of 0.6–1.0 nm, with all particles being below 1 nm. The high dispersion an ultra-small size of the Pt-RE SBCs are advantageous for catalytic performance, as they provide a high density of active sites and facilitate strong metal–support interactions. The EDS spectra (Figure 3c–e) clearly show the uniform distribution of Pt and Sc elements in the catalyst.

**Figure 4:**
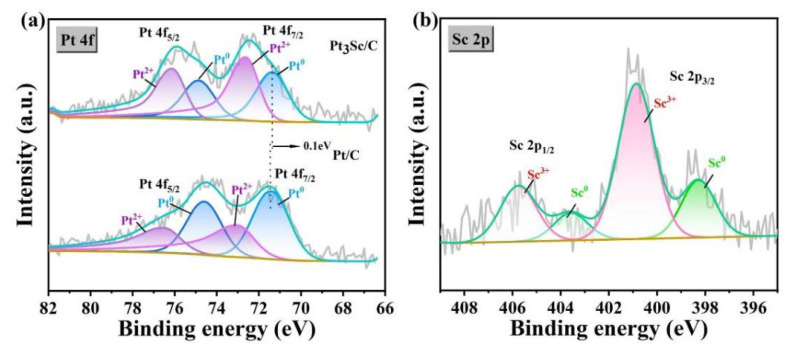
(**a**) Pt 4f XPS spectra of Pt-Sc/C and Pt/C, (**b**) Sc 2p XPS spectra of Pt-Sc.

The electronic structure of Pt and Sc in Pt-Sc SBCs was studied by XPS to elucidate the electronic interaction between the two elements and its effect on catalytic performance. Figure 4a displays the Pt 4f XPS spectra and corresponding deconvolution results of Pt-Sc and Pt/C. The fitting reveals that binding energy of the Pt 4f_7/2_ in the metallic state of Pt-Sc is approximately 0.1 eV lower than that of in Pt/C, indicating electron transfer from Sc to Pt in the SBCs. The presence of both metallic and oxidized states of Sc further supports the occurrence of electron transfer from Sc to Pt. This charge redistribution can be attributed to the significant electronegativity difference between Pt (2.28) and Sc (1.36), which weakens the electron binding in the Pt 4f orbital. Such electronic modulation is likely to lower the d-band center of Pt, potentially enhancing its catalytic activity.

**Figure 5:**
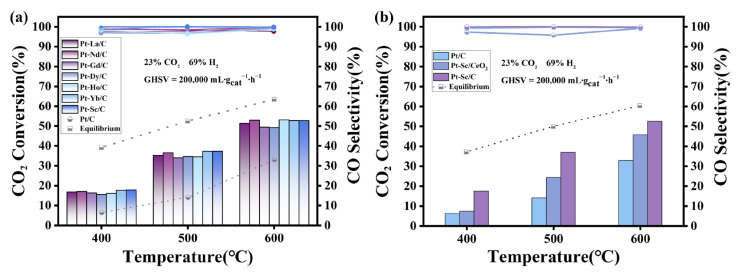
(**a**) The CO_2_ conversion and CO selectivity of the catalysts were investigated. (**b**) The CO_2_ conversion and CO selectivity of Pt-RE on different carriers.

The catalytic performance of the synthesized Pt-RE SBC series was evaluated to determine the effect of SBCs. Figure 5a shows the CO_2_ conversion and CO selectivity. The Pt-RE SBC catalysts exhibit excellent catalytic performance in the RWGS reaction. Compared with the conventional Pt/C, the CO_2_ conversion of Pt-RE catalysts occurs approximately at 500 °C, while maintaining ultra-high CO selectivity between 96% and 100%. Among them, Pt-Sc showed the best performance, achieving a CO_2_ conversion of 37.28% with a CO selectivity of 99.97%. The electronegativities of Pt and Sc are 2.28 and 0.95, respectively. Due to the significant difference in electronegativity, electron transfer from Sc to Pt occurs in Pt-Sc clusters. As indicated by XPS, the lower Pt 4f_7/2_ binding energy in Pt-Sc and the presence of both metallic and oxidized Sc suggest electron transfer from Sc to Pt. The electron transfer process induced by the electronegativity difference effectively modulates the charge density and local electronic structure of the Pt active center, typically resulting in a downshift of its d-band center [42]. This modulates the charge density of Pt atoms, reducing the bond energy between the catalyst surface and adsorbates, thereby increasing CO_2_ conversion and CO selectivity [35,36]. The presence of oxidized Sc on the surface also introduces a Pt-Sc_2_O_3_ interface, which promotes CO_2_ activation and dissociation (C-O bond cleavage). This interface effect not only improves CO_2_ conversion, but also directs the reaction pathway to favor CO production, significantly suppressing methane formation by inhibiting over-hydrogenation [12,19]. Under the reaction conditions of 500 °C, the synthesized series of Pt-RE SBCs exhibit slightly different CO_2_ catalytic conversion performance, and the order is as follows: Pt-Sc (37.28%) > Pt-Yb (37.24%) > Pt-Nd (36.51%) > Pt-La (35.29%) > Pt-Ho (34.58%) > Pt-Dy (34.45%) > Pt-Gd (33.97%).

To examine the effect of support on Pt-RE SBCs catalysis, Pt-Sc was synthesized on different supports and tested by in RWGS reaction. Figure 5b compares the CO_2_ conversion and CO selectivity of Pt-Sc on different supports. Both Pt-Sc/CeO_2_ and Pt-Sc/C show improved CO_2_ conversion compared to Pt/C, along with high CO selectivity (95–100%), underscoring the beneficial role of Sc doping in Pt-based catalysts. However, Pt-Sc/CeO_2_ exhibits lower CO_2_ conversion than Pt-Sc/C. Figure S3b,c shows the particle size distribution of Pt-Sc/C and Pt-Sc/CeO_2_, respectively. Statistical analysis indicates that when CeO_2_ is used as the support, the Pt-Sc nanoparticles exhibit a larger average size and a broader size distribution. The increased particle size likely reduces the number of active sites and lowers the surface energy, thus affecting the catalytic performance. Although the oxygen storage/release capacity of CeO_2_ may facilitate CO_2_ activation, the larger particle size (15.95 nm) limits the number of active sites, which may be the main reason for the lower activity enhancement for Pt-Sc/CeO_2_. Moreover, larger metal particles are more prone to sintering, which can further compromise catalytic activity and stability.

**Figure 6:**
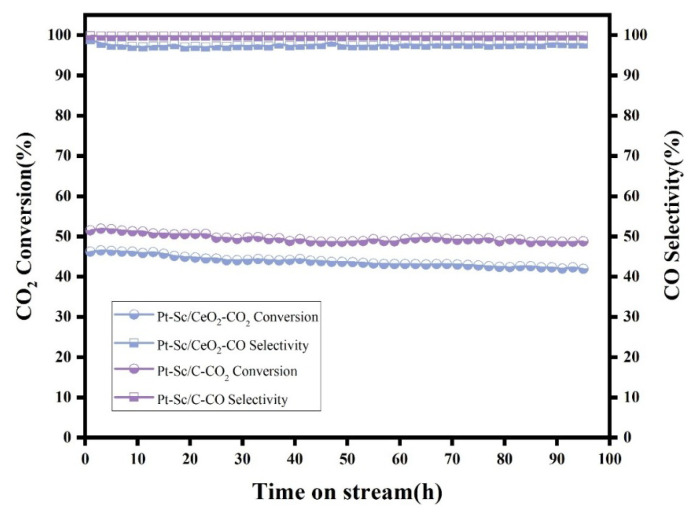
Stability test of Pt-Sc/C and Pt-Sc/CeO_2_ at 600 °C for 100 h.

The stability of the catalysts was evaluated at 600 °C, and the results are presented in Figure 6. Both Pt-Sc/C and Pt-Sc/CeO_2_ show excellent stability. The CO selectivity of both samples remained nearly unchanged over 100 h, while the CO_2_ conversion remained relatively stable. After 100 h, the CO_2_ conversion of Pt-Sc/C decreased by approximately 3.3%, and that of Pt-Sc/CeO_2_ decreased by about 4.3%. The high stability of Pt-Sc SBCs can be attributed to the strong metal–metal bond energy between Pt and Sc induced by the high formation energy of the Pt-Sc alloy. Due to the high thermal stability of noble metal–rare earth alloys, it can maintain structural integrity in the reaction environment at 600 °C, thus directly inhibiting the migration and agglomeration of clusters. The rare earth component optimizes the electronic structure of Pt through electronic effects and directly participates in the anchoring and activation of oxygen atoms in CO_2_ via its inherent Lewis acidity or oxygen storage/release capacity. This bifunctional or multifunctional synergy enables the originally inert linear CO_2_ molecule to undergo bending and dissociation with a lower energy barrier, thereby being efficiently converted into the target product [42]. The role of platinum lies primarily in efficiently initiating H_2_O dissociation, mediating the redox cycle, and optimizing the adsorption equilibrium of reaction intermediates through its tunable electronic structure [43]. The incorporation of rare earth elements primarily strengthens the adsorption of CO_2_ molecules on Pt and enhances the direct cleavage of C=O bonds through electronic and geometric effects, rather than shifting toward the formate pathway requiring sequential hydrogenation. These clusters tend to occupy high-energy defect sites on the support surface. This interaction resembles “atomic pinning,” anchoring the metal atoms at specific locations, which significantly increases the activation energy for their surface migration and prevents their movement and aggregation even under high-temperature conditions. Highly conductive carbon materials can donate a small number of electrons to Pt, forming electron-enriched Pt. This helps weaken CO adsorption and prevents CO poisoning.

In bimetallic catalyst systems, the reaction mechanism primarily depends on either “spatial functional separation and coupling” or “electronic and structural modulation at the atomic level.” Both are effective strategies for enhancing the RWGS performance of Pt-based catalysts, yet they correspond to fundamentally different characteristics of active sites, microscopic reaction pathways, and principles of catalyst design. In practical investigations, these two mechanisms may even coexist or compete on the same catalyst [44,45].

## 4. Conclusions

In this study, a series of Pt-RE SBCs catalysts with high activity, selectivity, and stability were successfully developed for a RWGS reaction. The introduction of rare earth elements effectively regulates the electronic structure of Pt, leading to enhanced CO_2_ conversion and suppression of CO methanation. The influence of different supports on the catalytic performance of Pt-RE SBCs was also investigated. Compared with CeO_2_, carbon supports promote more uniform dispersion and the formation of ultra-small SBCs, thereby improving catalytic performance. This work provides new insights into the design of efficient and stable RWGS catalysts, highlighting the key role of the synergistic effect at the Pt-RE SBC interface and importance of support selection in CO_2_ utilization. Rare earth resources are limited and precious. Therefore, a key research objective is to reduce the usage of rare earth elements without compromising catalytic performance.

## Data Availability

All data needed to support the conclusions in the paper are presented in the manuscript and/or the Electronic Supplementary Material. Additional data related to this paper may be requested from the corresponding author upon request.

## Supplementary Materials

The following supporting information can be downloaded at https://www.mdpi.com/article/10.3390/nano16010077/s1, Figure S1: The EDS elemental mapping images of the catalysts (a–c) Pt-La/C, (d–f) Pt-Nd/C, (g–i) Pt-Gd/C, (j–l) Pt-Dy/C, (m–o) Pt-Ho/C, (p–r) Pt-Yb/C used in this study; Figure S2: XRD patterns of Pt-Sc/C and Pt-Sc/CeO_2_; Figure S3: TEM images of (a) Pt/C, (b) Pt-Sc/C with illustrations of size distribution, (c) Pt-Sc/CeO_2_ with illustrations of size distribution. EDS elemental spectra of the catalysts (d–f) Pt-Sc/C, (j–i) Pt-Sc/CeO_2_.
