# Supplementary material for: Pt-Rare Earth Subnanometric Bimetallic Clusters Efficiently Catalyze the Reverse Water–Gas Reaction
Source: Nanomaterials (Basel). 2026 Jan 5;16(1):77. doi: 10.3390/nano16010077 (PMC12787743; doi:10.3390/nano16010077)
Supplement: Supplementary file 1 [file nanomaterials-16-00077-s001.zip › nanomaterials-4040213-supplementary.pdf]

Pt-rare earth subnanometric bimetallic clusters efficiently catalyze the reverse water gas reaction

Zhaolei Liang [1], Chang Sun [1] Songhe Shen [2] Qingqing Li [3,*] and Feng Luo [1,*]

[1] Tianjin Key Lab for Rare Earth Materials and Applications, Center for Rare Earth and Inorganic Functional Materials, School of Materials Science and Engineering, National Institute for Advanced Materials, Nankai University, Tianjin 300350, China; liangzhaolei@mail.nankai.edu.cn(Z.L.); sunchang@mail.nankai.edu.cn(C.S.)
[2] Department of Physics, University of California at Santa Barbara, Santa Barbara CA 93106, USA; shen16052061@gmail.com(S.S.)
[3] College of renewable energy, Hohai University, Nanjing 211100, China
* Correspondence: liqingqing@hhu.edu.cn(Q.L.); feng.luo@nankai.edu.cn(F.L.)

To prepare CeO_2 polyhedra, 19.2g NaOH and 1.736g $Ce(NO_3)_2·6H_2O$ was dissolved in 80 ml of ultrapure water. Place the solution in a hydrothermal reactor and react at 100 °C for 24 hours, The precipitate was washed with water until neutral, vacuum dried, and calcined at 350 °C in air for 4 hours.

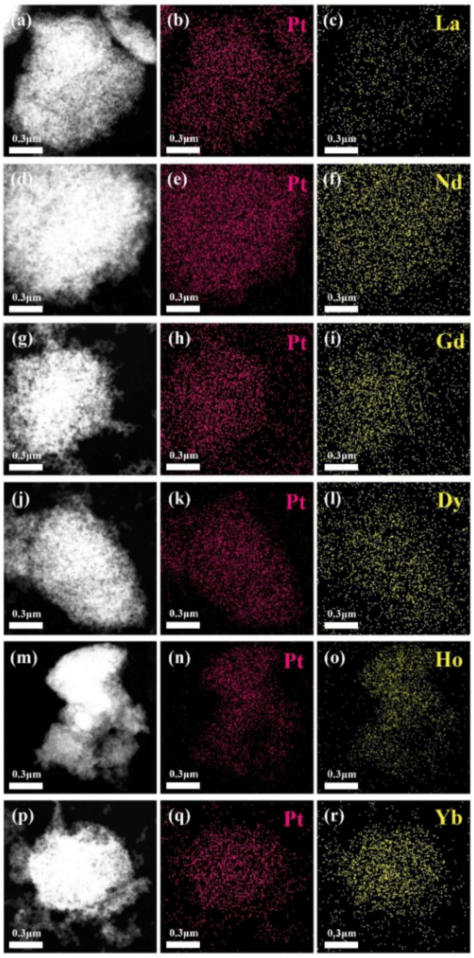

Figure S1. The EDS elemental mapping images of the catalysts (a-c)Pt-La/C; (d-f)Pt-Nd/C; (g-i)Pt-Gd/C; (j-l)Pt-Dy/C; (m-o)Pt-Ho/C; (p-r)Pt-Yb/C used in this study.

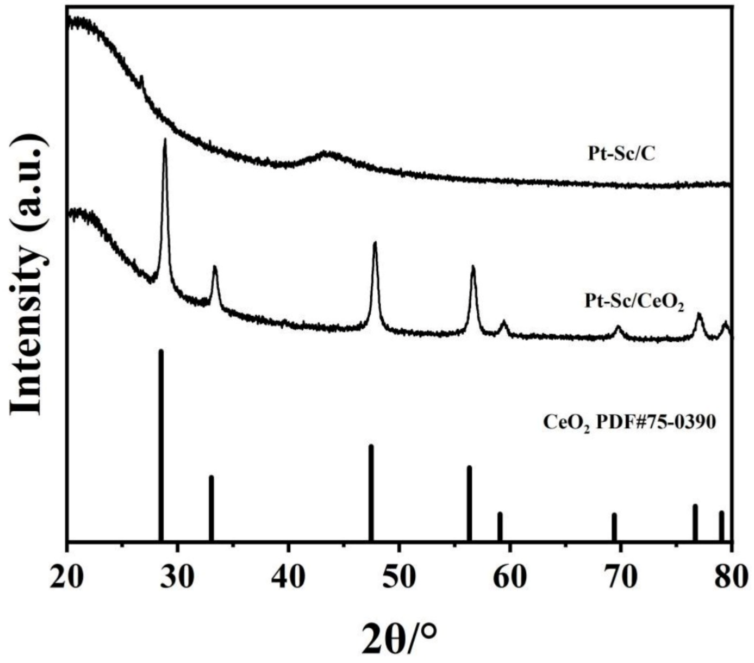

Figure S2. XRD patterns of of Pt-Sc/C and Pt-Sc/CeO_2.

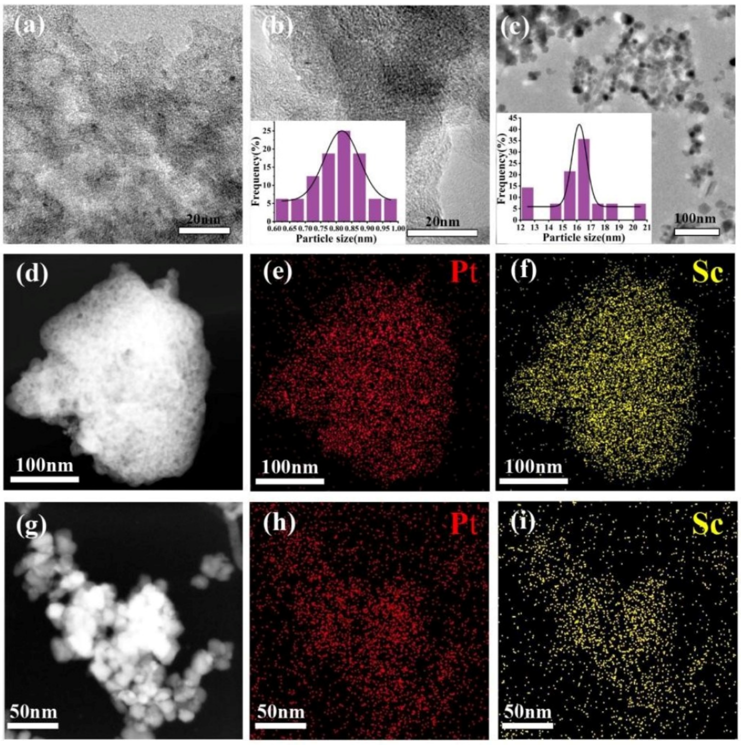

Figure S3. TEM images of (a) Pt/C, (b) Pt-Sc/C with illustrations of size distribution, (c)Pt-Sc/CeO_2 with illustrations of size distribution. EDS elemental spectra of the catalysts (d-f)Pt-Sc/C, (j-i)Pt-Sc/CeO_2.
